# Editorial: Public Health Matters: Pandemic, Policies, Processes

**DOI:** 10.3389/fpubh.2022.922652

**Published:** 2022-05-09

**Authors:** Noor Hazilah Abd Manaf, Yong Kang Cheah, Noor'ain Mohamad Yunus

**Affiliations:** ^1^Faculty of Economics and Management Sciences, International Islamic University Malaysia, Selayang, Malaysia; ^2^School of Economics, Finance and Banking, Universiti Utara Malaysia, Sintok, Malaysia; ^3^Faculty of Business and Management, Universiti Teknologi MARA, Puncak Alam, Malaysia

**Keywords:** COVID-19, disease, government, pandemic, public health

COVID-19 has shown that governments and public health are intertwined. The decisions and actions of one bear consequence on the other. Never has the role of government been more significant in public health than in this pandemic. Government policies directed toward controlling COVID-19 brought to fore the significance of public health, and they are not only on the health of the population, but on the wider socio-economic repercussions to the society. The governments mandated lockdowns shuttered businesses, resulting in massive job losses and ability of people to work. School closures, transportation restrictions and movement control, for instance, brought about other unintended economic and social consequences. Vaccine mandates touches more than just equity and access, but also on individual right and trust in the governments and the big pharma (Roberts et al.). Thereby, what is the right trajectory in pandemic management? Should governments opt for suppressive lockdowns or engage in individual compliance instead? Two years down the line, how did the policies and processes fare in managing COVID-19 and in extenuating its socio-economic fallout? This topic thus explores national policies and organizational processes in pandemic control and response across the globe, including China, Sweden, Kuwait, and Mali, as no countries were spared from the impacts of COVID-19.

A mathematical model developed by Al-Shammari et al. to study the impact of strict public health measures in Kuwait, such as closure of schools, government offices and non-essential businesses, and full full-border lockdown. The authors showed that these measures had a desirable effect on lowering the intensity of outbreak in the initial stage. Since Kuwait has a large number of migrant worker population living in cramped conditions making social distancing untenable, this further exacerbate transmission when a strict lockdown was implemented.

Saudi Arabia was proactive in its pandemic response, and was among the first few countries to close borders and shift formal education to online learning. Mixed method analysis by Alonazi and Altuwaijir supported the effectiveness of Saudi Arabia's COVID-19 containment policies, which were carried out through three domains: health guidelines, utilizing simulation such as, telehealth and telecommunication services, and ensuring continuity of services. Alsharqi et al., on the other hand, assessed inequalities in knowledge of COVID-19 in Saudi Arabia and found the existence of socioeconomic inequality in obtaining proper knowledge about the disease. Knowledge of COVID-19 was found to be more concentrated among the better educated and wealthier strata of the society, prompting the need for ensuring an effective dissemination of health information across social classes.

Fear of the virus coupled with strict public health measures caused disruption in economies around the world. To offset the shock, many governments introduced financial interventions and other safety measures to cushion the economic impact of the pandemic. In Mali, the government introduced COVID-19 Urban Cash Intervention (CUCI). The impact of this intervention and other safety measures was analyzed by Mnyanga et al. The authors showed that the intervention had a positive impact on households given that households were less likely to reduce food consumption, and rely on savings.

Fredriksson and Hallberg conducted a qualitative study related to COVID-19 testing in Sweden. The authors claimed that the independent public agencies and self-governing municipalities played an important role in providing understanding of COVID-19 testing. Their findings showed that having a mass test for COVID-19 in the country was a new issue which involved with numerous national and regional key players. This study also highlighted the importance of multi-level governance in managing healthcare during the crisis of COVID-19.

A case study about management of natural disaster and COVID-19 pandemic in Taiwan has been conducted by Wang et al. The authors argued that management for natural disasters could be used as a guideline to improve management for communicable diseases, such as COVID-19. Several important findings were highlighted by the authors: (1) government needed more financial resources and training to control COVID-19 pandemic; (2) a more flexible emergency operation for COVID-19 was needed; and (3) risk governance should play a more important role in controlling the spread of COVID-19.

Using data of Shandong and Zhejiang, Xu et al. examined the effects of hesitancy and region on use of COVID-19 vaccines. The authors found that parents in Zhejiang were more likely to use COVID-19 vaccines than those in Shandong. Factors influencing the hesitancy of parents included parental attitudes toward childhood vaccines, behavior as well as safety and efficacy of vaccines. Therefore, the authors suggested that strategies directed toward increasing use of COVID-19 vaccines among adolescents in Zhejiang and Shandong should take into account these factors.

Gavurora et al. made use of data of Slovak Republic to investigate alcohol consumption among adults during COVID-19 lockdown. The determining factors of excessive alcohol consumption were the main focus of this study. The authors found that the majority of people reduced their intake of alcohol during COVID-19 lockdown. In addition, findings of this study showed that the likelihood of excessive drinking was higher among males, younger adults and smokers compared with others. As pointed out by the authors, effective and evidenced based intervention measures could be implemented.

Drawing from Malaysian data, Cheong et al. analyzed spatiotemporal variations in the COVID-19 pandemic. Daily cumulated cases of COVID-19 were used for analyses. The authors found that risk of spreading COVID-19 increased across West and East Malaysia. In particular, the state with the most spread of COVID-19 was Melaka, followed by Sabah. Findings of this study could serve as useful guideline for COVID-19 prevention.

An article from Indonesia described the elements that influenced the inclusion of disabled workers during pandemics. According to the article, workers with disabilities were more vulnerable, which had an impact on their level of acceptance and inclusion at work (Ayuningtyas et al.). Another study used a survey approach to analyse public trust in the government of Bangladesh (GoB) and the private sector concerning the COVID-19 pandemic risk management. Findings from the study demonstrated that the GoB failed to satisfy public satisfaction levels regarding the food delivery and financial support among low- and middle-income people. This was due to a lack of collaboration and coordination among government and stakeholders (Alam et al.). There was a study using a mixed-method approach to examine the perceptions and attitudes linked with COVID-19 vaccination acceptability in the United Kingdom. Individual behaviors and the transparency of the vaccine development process were identified to be determining factors of people's willingness to participate in the COVID-19 vaccination programs (Roberts et al.).

## Author Contributions

NA, YC, and NM drafted the editorial. YC revised and approved the final version. All authors contributed to the article and approved the submitted version.

## Conflict of Interest

The authors declare that the research was conducted in the absence of any commercial or financial relationships that could be construed as a potential conflict of interest.

## Publisher's Note

All claims expressed in this article are solely those of the authors and do not necessarily represent those of their affiliated organizations, or those of the publisher, the editors and the reviewers. Any product that may be evaluated in this article, or claim that may be made by its manufacturer, is not guaranteed or endorsed by the publisher.

